# Beyond the classic GPCR: unraveling the role of GPR155 in cholesterol sensing and signaling

**DOI:** 10.1038/s41392-024-02059-w

**Published:** 2024-11-18

**Authors:** Torsten Schöneberg

**Affiliations:** 1 Rudolf Schönheimer Institute of Biochemistry, Molecular Biochemistry, Medical Faculty, University of Leipzig, Leipzig, Germany; 2 School of Medicine, University of Global Health Equity, Kigali, Rwanda

**Keywords:** Structural biology, Structural biology

In a recent cryo-EM study published in *Nature*, Bayly-Jones and co-workers have provided detailed molecular insights into the dimerization and cholesterol binding of the orphan G protein-coupled receptor GPR155, its potential involvement in gut microbiota-derived tryptophan metabolite interactions, and intracellular signaling pathways.^1^ GPR155, a lysosomal protein with a unique 17-transmembrane helix domain structure, integrates auxin transporter and G protein-coupled receptor-like features, playing a key role in lysosomal cholesterol sensing and mTORC1 signaling.

Our textbook understanding of G protein-coupled receptors (GPCRs) is shaped by the hallmark 7-transmembrane helix domain (7TMD), where the short N-terminus extends into the extracellular space and the C-terminus into the cytosol. Most GPCRs follow this structural organization, though there are exceptions. For instance, the glycoprotein hormone family, and classes of glutamate, adhesion, and secretin receptors have notably large N-termini with additional domains. Sequence analyses have also revealed GPCR transcript fusions with neighboring genes (e.g., PPAN-P2RY11) as well as membrane proteins with more than seven transmembrane helices (e.g., GPR89, GPR128, GPR155), initially classified in the GPCR superfamily due to sequence homology.^2^

**Fig. 1:**
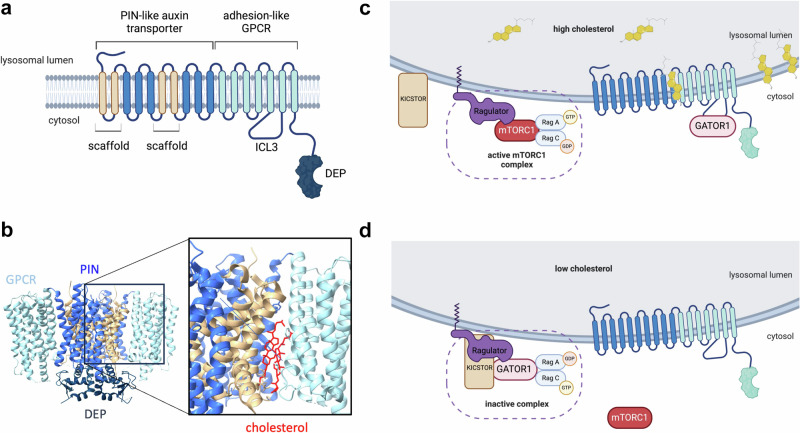
Topology, cryo-EM structure and signaling of GPR155. **a** Topology and schematic illustration of the GPR155 domain layout. **b** Cryo-EM studies revealed a homodimer assembly of GPR155.^1,5^ The three-dimensional arrangement of transmembrane helices is depicted (PDB accession number: 8U54) and colored according to (**a**). Cholesterol molecules bound at the interface between the transporter and GPCR domains are shown in red (PDB accession number: 8U5C). **c** The current model suggests that in the presence of cholesterol, GPR155 undergoes a conformational change, enabling it to bind with the GTPase-activating protein complex GATOR1 by disrupting the interaction between GATOR1 and the KICSTOR complex. GATOR1 recruitment promotes Rag GTPase activation, leading to mTORC1 activation and its localization at the lysosome. **d** Upon cholesterol depletion, GPR155 cannot disrupt the association between GATOR1 and KICSTOR, resulting in inhibition of mTORC1 signaling (signaling schema adapted from *Science*,^4^ and generated with biorender)

One such outlier is GPR155, first discovered in 2003 and originally named PGR22, DEP.7, and DEPDC3. GPR155 encodes a protein with an unusual 17-transmembrane helix structure.^3^ This arrangement is conserved across various species, from insects to vertebrates. In humans, GPR155 lacks a signal peptide but has a conserved region (amino acids 39–360) spanning ten transmembrane helices (TM1–10), which is similar to membrane transport and auxin efflux carrier proteins of the plant PIN family (Fig. 1a). Auxins, such as indole-3-acetic acid (IAA), are crucial phytohormones that regulate plant growth and development. The remaining seven transmembrane helices (TM11–17) of GPR155 share motifs with adhesion/secretin class B GPCRs, and the C-terminus includes a Dishevelled, Egl-10, and Pleckstrin (DEP) domain (Fig. 1a), which is common in signaling proteins like Regulator of G-protein Signaling (RGS) proteins that enhance GTPase activity.

In 2022, Shin et al. identified GPR155 as a lysosomal membrane protein functioning as a cholesterol sensor, regulating mTORC1 signaling.^4^ They named it LYCHOS (Lysosomal Cholesterol Signaling) protein. Until recently, the structural assembly and molecular function of GPR155 remained elusive. Now, two independent groups have provided monomeric and homodimeric structures determined via cryogenic electron microscopy (cryo-EM), shedding light on its cholesterol sensing and signaling functions.^1,5^

The dimerization interface of GPR155 is formed by conserved transmembrane domain (TM) interactions and less extensively by the DEP domains.^1^ Cholesterol binds at the interface between the transporter-like and GPCR domains (Fig. 1b) and mutations at this site lead to dysregulation of mTORC1 activation.^1,5^ Cholesterol homeostasis is vital for cellular membrane integrity, steroid hormone synthesis, and various signaling pathways. Lysosomes can sense cholesterol levels and their surface serves as a signaling platform. Cholesterol levels influence the GPR155-mediated recruitment of mTORC1 to lysosomes through interactions with the GTPase-activating protein complex GATOR1, leading to Rag GTPase activation^4^ (Fig. 1c, d). However, while cholesterol is often observed in structures of transmembrane proteins, the low resolution of the reported maps and the lack of functional validation still leave open the question of whether this cholesterol-binding site plays a direct role in cholesterol sensing.

Both groups also identified a common IAA binding site within GPR155’s transporter-like domain, though they presented conflicting data on its role as an IAA transporter. The outward-open state of the transporter domain remains unresolved.^1,5^ In humans, IAA and other indole derivatives are produced by gut microbiota as tryptophan metabolites, potentially playing key roles in gut microbiome-host interactions. The unique combination of a transporter domain and a GPCR within one transmembrane assembly suggests a functional link between transport and cholesterol sensing, with experimental evidence that this transport is necessary for cholesterol entry and subsequent mTORC1 regulation.^1^

However, a central question remains: Is GPR155 truly a G protein-coupled receptor? Its topology aligns the N-terminus of the GPCR domain inside the lysosomal lumen and the C-terminus on the cytosolic side, placing the potential G-protein interaction surface correctly toward cytosolic G proteins. Both structural studies confirm that the 7TMD shares the highest homology with adhesion/secretin class GPCRs. Adhesion GPCRs are among the oldest GPCRs, present in amoebozoa, fungi, and animals, suggesting that the 7TMD might have served as a modular domain fused with other transmembrane assemblies during bilaterian evolution. Adhesion GPCRs often feature modular N-termini, and at least one member, GPR128/ADGRG7, has an extended transmembrane topology beyond the classic 7TMD.^2^ Typically, adhesion GPCRs activate G-protein signaling through mechanical activation (via an internal agonist called the *Stachel* sequence). However, GPR155 lacks the GAIN domain, GPS, and *Stachel* sequences, suggesting that if it activates G-protein signaling, it likely follows a different mechanism. Moreover, several adhesion GPCRs have sterol derivatives as ligands; however, the cholesterol molecules observed in the GPR155 structures are located at the interface between the receptor and the transporter (Fig. 1b) and not in the orthosteric binding site, as seen, for example, with cortisol in GPR97/ADGRG3 (PDB accession number: 7D77).

The 7TMD of GPR155 could potentially bind ligands, but the orthosteric site is blocked by the lysosomal lumen-facing loop 7, homologous to the second extracellular loop (ECL2) of typical GPCRs. Many GPCRs feature a disulfide bond linking ECL1 and ECL2, crucial for their function, but this bond is absent in GPR155. GATOR1 binds to the cytosol-facing loop 7 (homologous to the ICL3 in other GPCRs) and localizes to the lysosomal membrane, enabling mTORC1 activation. However, GPR155’s large and dynamic ICL3 remains unresolved structurally, leaving questions about how its interaction with GATOR1 is regulated by cholesterol. Furthermore, the DEP domain, commonly found in RGS proteins, interacts with the Gβγ subunit of heterotrimeric G proteins, though whether such interactions occur in GPR155 remains unknown. Nevertheless, the 7TMD of GPR155 resembles canonical GPCRs, raising the possibility of agonist-mediated G-protein activation. Interestingly, disulfide bond-lacking GPCRs (e.g., melanocortin type 4 receptor), those with large, disordered ICL3 regions, (e.g., muscarinic acetylcholine receptors), and non-canonical signaling, such as ELMO1/DOCK1 activation by several adhesion GPCR, demonstrate that deviations from typical GPCR architecture do not preclude functionality. Additionally, there is growing evidence of GPCR signaling from intracellular compartments. Future research will explore these open questions, potentially adding new dimensions to the diverse intracellular repertoire of GPCR-mediated signals and pathways.
